# Levonorgestrel Microneedle Array Patch for Sustained Release Contraception: Formulation, Optimization and In Vivo Characterization

**DOI:** 10.3390/molecules27072349

**Published:** 2022-04-06

**Authors:** Amarjitsing Rajput, Riyaz Ali M. Osmani, Achyut Khire, Sanket Jaiswal, Rinti Banerjee

**Affiliations:** 1 Nanomedicine Lab, Department of Biosciences and Bioengineering (BSBE), Indian Institute of Technology Bombay (IITB), Mumbai 400076, India; khireachyut@gmail.com (A.K.); sanketvjaiswal@gmail.com (S.J.); rinti@iitb.ac.in (R.B.); 2 Department of Pharmaceutics, Poona College of Pharmacy, Bharti Vidyapeeth Deemed University, Pune 411038, India; 3 Department of Pharmaceutics, JSS College of Pharmacy (JSSCP), JSS Academy of Higher Education & Research (JSS AHER), Mysuru 570015, India

**Keywords:** drug delivery, nanotechnology, contraception, levonorgestrel, liposomes, microneedle, sustained-release

## Abstract

Background: The goal of this work was to develop a levonorgestrel liposome-loaded microneedle array patch for contraception. Methods: Levonorgestrel-loaded liposome was formulated by a solvent injection technique, characterized, and studied. Results: The formulated liposomes were characterized for particle size (147 ± 8 nm), polydispersity index (0.207 ± 0.03), zeta potential (−23 ± 4.25 mV), drug loading (18 ± 3.22%) and entrapment efficiency (85 ± 4.34%). A cryo high-resolution transmission electron microscopy and cryo field emission gun scanning electron microscopy study showed spherical shaped particles with a smooth surface. The in vitro drug release and in vivo pharmacokinetic study showed sustained behaviour of Levonorgestrel for 28 days. Conclusion: The levonorgestrel liposome-loaded microneedle array patch showed better contraception than the drug-loaded microneedle array patch.

## 1. Introduction

Despite advances in contraception methods available in the market, 67 million pregnancies were unplanned throughout the world in 2017 [1]. The number of pregnancies accounts for around 56 million abortions every year [2]. Over 40% of births are unwanted worldwide, with approximately 20% of those ending in abortion [3]. This high prevalence of unwanted pregnancy leads to high economic and social burdens for women. The reasons for unintended pregnancy include limited access to contraception, younger populations, poverty, unmarried people, side effects associated with contraceptive methods, cultural or religious conflicts, poor-quality contraceptive agents, preferences of users and providers, and gender-associated barriers. In the world, 62% of married women (age 15 to 49) use family planning procedures and modern methods are used by 56% of the population [4]. Many methods are currently available, such as combined oral contraceptives (COCs) or the pill, progestogen-only pills (POPs), implants, progestogen-only injectable devices, monthly injectable devices or mixed injectable contraceptives (CIC), mixed contraceptive patches, mixed contraceptive vaginal rings (CVRs), intrauterine devices containing copper (IUDs), intrauterine device loaded with levonorgestrel (IUDs), male and female condoms, male sterilization (vasectomy), female sterilization (tubal ligation), lactational amenorrhea method (LAM), contraception pills for emergency use (composition: ulipristal acetate 30 mg or levonorgestrel 1.5 mg), the standard days m (SDM), basal body temperature (BBT) method, two day method, sympto-thermal method, etc. Traditional methods are also used for contraception, such as the calendar method or rhythm method and withdrawal (coitus interruptus) [3]. These are effective contraception methods, but some require frequent dosing; as a result, they suffer from poor patient compliance [5]. Modern contraception methods need healthcare professionals for administration, hence are not suitable for people in low-income countries [6]. Thus, it is essential to formulate a safe, cost-effective, long-acting, self-administrable and patient-acceptable contraceptive-based system for global use [7,8,9].

Liposomes are vesicular structures that consist of a lipid bilayer enclosed by an aqueous membrane. Liposomes are widely used as lipid-based nanocarrier systems for both hydrophilic and hydrophobic compounds [10]. They have shown significant enhancement in the delivery and therapeutic potency of different classes of active pharmaceutical ingredients. The effective delivery of liposomes improves various drugs’ pharmacodynamic and pharmacokinetic properties [11,12].

Microneedles are micrometer-size structures that penetrate through the skin layer, not to the dermis, to promote the passage of the drug across the skin layers without causing pain [13,14,15,16]. Microneedles are an economical and suitable dosage form for self-administration by the patient. They are made of biocompatible and biodegradable materials and release the drugs slowly. An incredible, extremely unique, effective and minimally invasive development has been achieved in the era of microneedle technology to improve intradermal and transdermal delivery of different medications [17,18,19,20,21,22]. Microneedles are considered as a viable approach for the immediate and long-acting delivery of drugs across the skin [23]. Therefore, microneedles are drawing more attention from researchers and clinicians [24].

In this study, we developed and characterized a contraceptive hormone (levonorgestrel, LNG) liposome-based microneedle array patch in terms of different parameters such as microscopy, scanning electron microscopy (SEM), in vitro skin piercing, in vitro skin penetration, mechanical strength, etc. Finally, we investigated in vitro drug release and in vivo pharmacokinetics in rats for 28 days. The aims of the study were to (i) reduce the dosing frequency, (ii) avoid side effects associated with frequent administration of other contraceptive agents, and (iii) provide sustained drug release for 28 days.

## 2. Materials and Methods

### 2.1. Materials

Levonorgestrel was purchased from Cayman Chemicals, Bangalore, India. Soya phosphatidylcholine (SPC) and oleic acid were gifted and purchased from VAV life sciences, Mumbai, India, and Merck India Ltd., Mumbai, India, respectively. Gelatin and polyvinyl alcohol were purchased from MP Biomedical, Mumbai, India. Polydimethylsiloxane (PDMS) and elastomer were purchased from DuPont, Mumbai, India. All chemicals and reagents used for the study were analytical grade. All solvents used for analytical study were HPLC-grade.

### 2.2. Methods

#### 2.2.1. Preparation of Liposomes

Levonorgestrel liposomes were formulated by the solvent injection method, as reported by Goudo et al. [25]. Weighed quantities of soya phosphatidylcholine and oleic acid were dissolved in ethanol. Simultaneously, a weighed amount of phosphate buffer solution (PBS, pH 7.4) was subjected to stirring on a magnetic stirrer (Remi, MLV, Mumbai, India). After that, the ethanolic lipid solution was injected into the phosphate buffer solution (pH 7.4) using a 20-gauge needle (dimensions: 0.9 mm × 25 mm) dropwise to form multilamellar vesicles and allowed to stir for 1 h at 1000 rpm. The formed multilamellar vesicles (MLVs) were further processed using high-speed homogenization (IKA, T50 Digital Ultra Turrax, Ahmedabad, India) at 10,000 rpm for 2 min. Finally, large unilamellar vesicles (LUVs) were subjected to stirring for 24 h to remove the solvent.

#### 2.2.2. Characterization of Liposomes

##### Measurement of Particle Diameter and Zeta Potential

The prepared liposomes were characterized for particle size, polydispersity index, and zeta potential using a particle size measurement instrument based on the dynamic light scattering (DLS) principle (Malvern Instruments, Zetasizer Nano ZS Ultra, Malvern, UK). The experiments were conducted in triplicate using 2 mL of the formulation at 25 ± 2 °C with a 90° scattering angle [26,27].

##### Determination of Drug Loading and Encapsulation Efficiency

Using the centrifugation technique, the formulated liposomes were further characterized for drug loading and encapsulation efficiency. A definite quantity of liposome (2 mL) was centrifuged (Beckman Coulter Inc., Optima XPN-100 Ultracentrifuge, Indianapolis, IN, USA) at 32,000 rpm with average g-force of 152535 (rotor type-swinging bucket SW 32 Ti, tube-polypropylene quick seal with 10 mL capacity) for 6 h at 4 °C to separate the free drug from the liposome. Then, the upper layer was separated, filtered through a 0.22 µ filter, and quantified by HPLC (Jasco, 4000, Tokyo, Japan) with a photodiode array (PDA) detector (Jasco, MD 4015, Tokyo, Japan). The drug loading and encapsulation efficiency were calculated using Equations (1) and (2), respectively [28,29].

(1) $$\mathrm{Drug}\;\mathrm{loading}=\frac{Total\;\;drug\;amount-Free\;drug\;amount}{Total\;lipid\;amount}*100$$

(2) $$\mathrm{Entrapment}\;\mathrm{efficiency}\;=\frac{Total\;drug\;amount-Free\;drug\;amount}{Total\;drug\;amount}*100$$

##### Cryo High-Resolution Transmission Electron Microscopy (Cryo HR TEM)

The shape and surface of the prepared liposomes were determined using a cryo high-resolution transmission electron microscope (Cryo HR TEM) (JEOL, JEM 2100, Tokyo, Japan). A single drop of liposome dispersion (10 µL) was kept on a copper grid coated with carbon and stained negatively using a phosphotungstic acid solution (1% (*w*/*v*)) and observed at 100 kV voltage [30].

##### Cryo Field Emission Gun Scanning Electron Microscopy (Cryo FEG SEM)

The surface structure of developed liposomes was determined using cryo field emission gun scanning electron microscopy (cryo FEG SEM) (JEOL, JSM-7600F, Tokyo, Japan). The liposome sample (5–10 µL) was placed on a carbon tape and lyophilized using liquid nitrogen. The images were captured by a cryo FEG SEM microscope, operated at 30 kV voltage with suitable magnification [31].

#### 2.2.3. Fabrication of Microneedle Array Patch

A master mold with metallic microneedles formed as an array was fabricated using the EDM facility at IDEMI, Mumbai. The diameter of the mold was 35 mm, and the needle dimensions were 900 μm height, 300 μm base diameter, and pitch of 1.5 mm, for an 18 × 18 array. To obtain a working mold consisting of needle cavities, polydimethylsiloxane (PDMS) compound (a mixture of elastomer and curing agent 10:1, mixed manually and degassed) was poured on the master mold and heated at 90 °C in the oven for 50 min. The cured compound was separated from the master mold and was ready for microneedle array patch fabrication. The PDMS molds were filled with PVA–gelatin mixture and LNG-loaded liposomes, all in defined proportions. After that, the molds were put onto the centrifuge’s plate rotor and centrifuged (Thermo Scientific, Fiberlite™ H3-LV Large Volume Swinging Bucker rotor, New York, NY, USA) for 45 min at 3000 rpm at g-force of 1840× *g*. After centrifugation, the mold was kept in the isolation chamber (at room temperature and 45% RH) for 24 h for drying. After drying, the microneedles were separated from the mold and characterized [16,32].

#### 2.2.4. Characterization of LNG Liposome-Loaded Microneedle Array Patch

##### Microscopy Study

Microneedle array patch images were captured from different directions and angles using a stereomicroscope (Nikon, SZX2, Tokyo, Japan) to study the needles’ shapes, sizes, and arrangements in the array.

##### Field Emission Gun Scanning Electron Microscopy (FEG SEM)

The drug-loaded microneedle array patch was placed on an ultra-microtome, and a section of suitable size was used for the study. The microneedle array patch was placed on two-sided tape with a metal stub and sputter-coated (10 nm thick) with Au/Pd and studied with a field emission gun scanning electron microscope (JEOL, JSM-7600F, Japan). The microneedle was examined for the drug in the microneedle [33].

##### In Vitro Drug Release

A USP type II dissolution apparatus was used to conduct an in vitro release evaluation of a liposome (containing LNG) loaded microneedle array patch. (Electrolab, TDT-08L, Navi Mumbai, India). For the release study, one microneedle array patch was suspended in 75 mL of phosphate buffer pH 7.4 (release medium) containing 0.1% *w*/*w* sodium azide at 37 °C at a speed of 50 rpm. Samples (2 mL) were removed and replaced with an equivalent volume of the release medium at various time intervals (1, 2, 4, 6, 8, 12, and every 24 h up to 28 days) to maintain sink condition. The amount of drug release was determined using an HPLC with a PDA detector [34].

##### Mechanical Strength Study

A universal testing machine (UTM) (Tinius Olsen, Inc., H5KS, Buskerud, Norway) was used to determine the mechanical strength of the LNG liposome-loaded microneedle array patch. Then, the patch was placed on a bottom circular plate workbench. Initially, the spacing between the microneedle array patch and the top circular plate was maintained at 3 mm. The upper workbench then slid down to the microneedle array patch at a 1 mm·min^−1^ pace. After that, the upper workbench moved down to the microneedle array patch at a speed of 1 mm·min^−1^. The testing machine determined the load and displacement values, and the weight-displacement curve was plotted to calculate the compression strength [33].

##### Skin Piercing Study

The strength desired for a liposome-loaded microneedle array patch for piercing into the skin was studied using properly shaved and excised skin of rat. The skin was subjected to 37 °C for 2 h prior to the study. The skin was kept on Styrofoam block, flattened, and maintained with the help of pins. The LNG liposome-loaded microneedle array was attached to a universal testing machine (UTM) probe with two-side tape. The probe was brought down onto the skin, allowed to penetrate the skin up to a certain distance (0.5 mm), and remained in site for 30 s. After that, the skin was treated with rhodamine dye solution for 30 s and imaged under an optical microscope [35,36].

##### Skin Irritancy Study

Before and after microneedle array patch insertion, skin integrity was studied on Wistar female rats weighing 200–250 g. The blank microneedle and microneedle array patch loaded with liposomes was applied to the dorsal part of the rat. The rat skin was observed for any reactions (i.e., redness, inflammation, or swelling) 24 h post application of the microneedle array patch [37,38].

#### 2.2.5. Statistical Analysis

The data were presented as means with standard deviation (n = 6). Microsoft Excel and GraphPad Prism (GraphPad Software Inc. La Jolla, CA, USA, trial version 5.0) were used to perform the statistical calculations [39].

## 3. Results and Discussion

### 3.1. Preparation of LNG Loaded Liposomes

Various lipids such as hydrogenated soy phosphatidylcholine (HSPC), distearoyl phosphatidylcholine (DSPC), dioleoyl phosphatidylcholine (DOPC), dipalmitoylphosphatidylcholine, egg phosphatidylcholine, and soy phosphatidylcholine were screened for the preparation of LNG loaded liposomes. The liposomes were prepared using the widely used ethanol injection method. The ethanol injection method is very useful for developing liposomes on a large scale. It is also an easy and continuous method [40]. The various parameters, such as syringe gauge, stirring speed, homogenization time, homogenization speed, and solvent quantity, were studied during preliminary trials (data not shown). It was found that 21 G syringe gauge, 1000 rpm stirring speed, 2 min sonication time, 10,000 rpm homogenization speed, and 2.5 mL of solvent were the optimal parameters for the formulation of liposomes with desired particle size and entrapment efficiency. During the preparation of liposomes, we used a perfusion automatic device or pump. The optimization was carried out using different injection speeds. The injection speed of 0.5 mL/min was considered suitable for preparation of uniform-size liposomes.

Finally, the liposomes prepared using soy phosphatidylcholine as a lipid showed the desired characteristics. Along with soy PC, oleic acid was also added to the formulation to promote the permeation of microneedles through the skin. Oleic acid was used as a permeation enhancer, which acts by modulating the extracellular lipids of the stratum corneum layer (the major barrier to skin permeation) of the skin [41]. It also decreases the resistance of the skin to diffusion by networking with the lipid matrix, leading to enhanced fluidity of the lipid [42,43]. The composition of all batches is shown in Table 1.

### 3.2. Characterization of Liposomes

#### 3.2.1. Particle Size, Polydispersity Index, and Zeta Potential

The particle size, polydispersity index, and zeta potential were determined by a method based on the zeta sizer’s dynamic laser light scattering mechanism. The average particle size and polydispersity index were measured by the equilibration time of 120 s for each sample using triplicate measurement. The particle sizes of batches L1, L2, and L3 increased with soy PC concentration, from 207 ± 4.21 to 245 ± 7.15 nm. The addition of oleic acid into the liposome formulation resulted in flexible liposomes with smaller particle sizes (Batch L4). The particle sizes of the L5 and L6 batches were found to be 169 nm and 157 nm, respectively, due to variation in the ratio of soy PC to oleic acid. The particle graph of optimized batch L6 is shown in Supplementary Materials Figure S1.

The polydispersity index shows how the particles in a formulation are distributed in size. The narrow particle size distribution suggested that the formulation had a homogeneous particle size distribution. The particle size distribution was <0.350 in all batches [44].

The colloidal dispersion stability was determined by measuring the zeta potential of the formulations. When the value of zeta potential increases, the attraction between the particles decreases, resulting in the enhancement of the stability of the formulation [45]. The impact of soy PC on the zeta potential was also studied. It suggested that as the concentration of soy PC increased, the zeta potential value also increased, as observed in batch L6 shown in Table 2. Batch L6 showed the highest zeta potential, of around −19 ± 4 mV.

#### 3.2.2. Encapsulation Efficiency

The encapsulation efficiency results for all batches are shown in Table 2. The batches were prepared using different drug: lipid ratios such as 1:5, 1:7, and 1:10 to determine their impact on entrapment efficiency. It was observed that as the drug: lipid ratio increased from 1:5 to 1:10, the encapsulation efficiency was enhanced, from 65.34 ± 5.44 to 85.24 ± 6.15. The increase in the quantity of lipids provided additional space for accumulating the drug and preventing the loss of the drug into the external phase. This may be due to an excess of lipid forming a layer around the particle. The superior imperfections in the crystal lattice of lipid provide adequate space to encapsulate drug particles [46,47,48].

The batches were also prepared with different ratios of soya PC: oleic acid, such as 5:5, 6:4, and 7:3. The increase in the quantity of lipids relative to the oleic acid resulted in higher entrapment efficiency, of around 85.24 ± 6.15 (Batch L6).

#### 3.2.3. Cryo High-Resolution Transmission Electron Microscopy (Cryo HR TEM)

**Figure 1:**
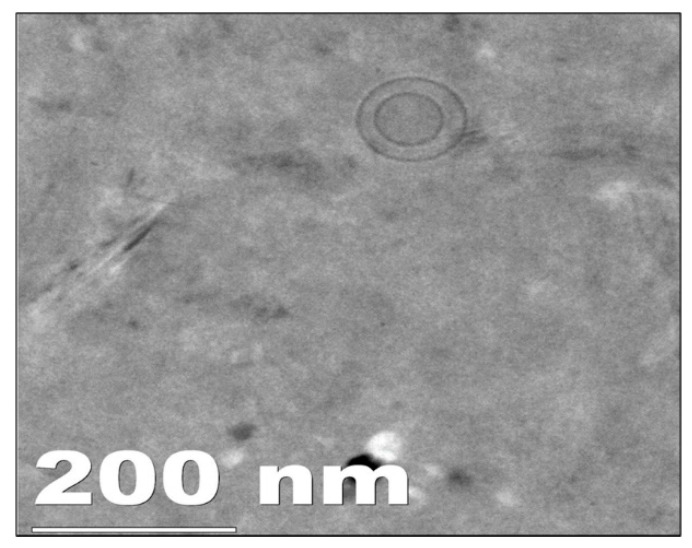
Cryo HR TEM image of optimized liposome (Batch L6).

The cryo HR TEM image of LNG-loaded liposomes showed a particle size of around 100 nm, as shown in Figure 1. The particle size obtained using dynamic light scattering was found to be 157 nm. This difference in the particle size obtained using both techniques was due to their different analysis mechanisms. The DLS technique determines the particle size by measuring the hydrodynamic diameter of the particle, whereas TEM determines the particle size with the particle mounted on the grid. The Cryo HR TEM image of liposomes showed a monodispersed unilamellar vesicle with a spherical shape [49].

#### 3.2.4. Cryo Field Emission Gun Scanning Electron Microscopy (Cryo FEG SEM)

**Figure 2:**
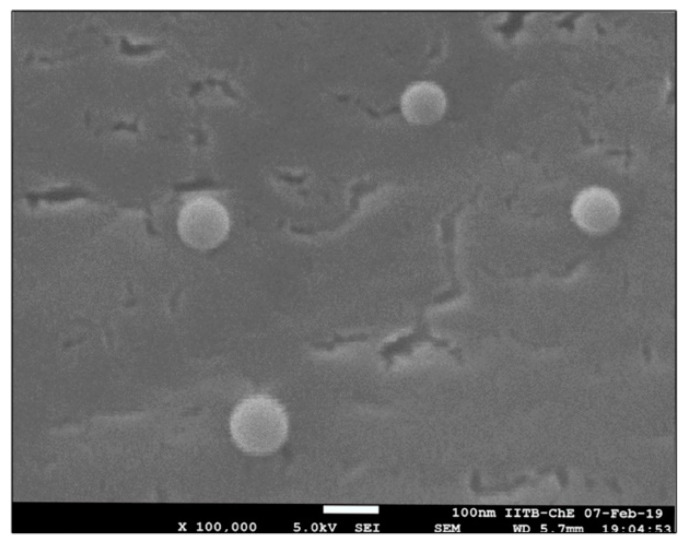
Cryo FEG SEM image of optimized liposome (Batch L6).

The cryo FEG SEM imaging study found liposomes containing LNG to be around 100–200 nm in size and spherically shaped with a smooth surface (Figure 2). The results were the same as those obtained by TEM analysis and the particle size measurements using DLS.

## 4. Fabrication of Microneedle Array Patch

The microneedle array patch was prepared using a combination of the polymer matrix (PVA: Gelatin). PVA and gelatin are considered safe, biocompatible, and biodegradable materials [50,51,52]. A combination of PVA: gelatin solution was utilized prepare the microneedles and increase their mechanical strength. The various microneedle array patches were designed using different ratios of PVA: gelation, such as 1:0.25, 1:0.5, and 1:1. The microneedles were characterized for mechanical strength and prepared using a 1:0.5 ratio showed the desired mechanical strength. Hence, a 1:0.5 ratio of PVA: gelatin was selected for further formulation development.

## 5. Characterization of LNG Liposome-Loaded Microneedle Array Patch

### 5.1. Microscopy Study

Microscopy-derived images of the microneedles (Supplementary Figure S2) showed needle-shaped needles with their arrangement with the base.

### 5.2. Scanning Electron Microscopy (SEM) Study

**Figure 3:**
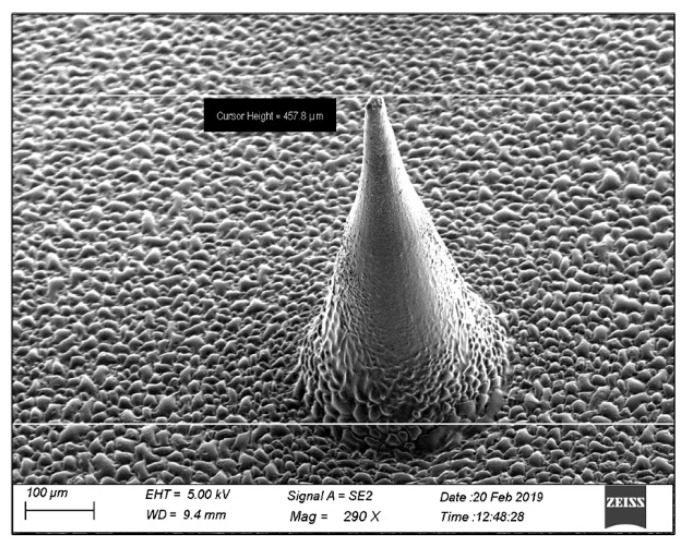
SEM image of levonorgestrel liposome-loaded microneedle.

Figure 3 shows a scanning electron microscopy image of the LNG liposome-loaded microneedle. The needle-shaped microneedle had an average length and a base diameter of 900 µm and 300 µm, respectively. The LNG liposome-loaded microneedle had a smooth surface without any cracks or fractures.

### 5.3. In Vitro Release Study

In the case of the dissolution of the microneedle array patch, the needle starts to dissolve in 15 ± 5 min, and complete dissolution of the microneedle array patch was observed within 35 ± 10 min.

**Figure 4:**
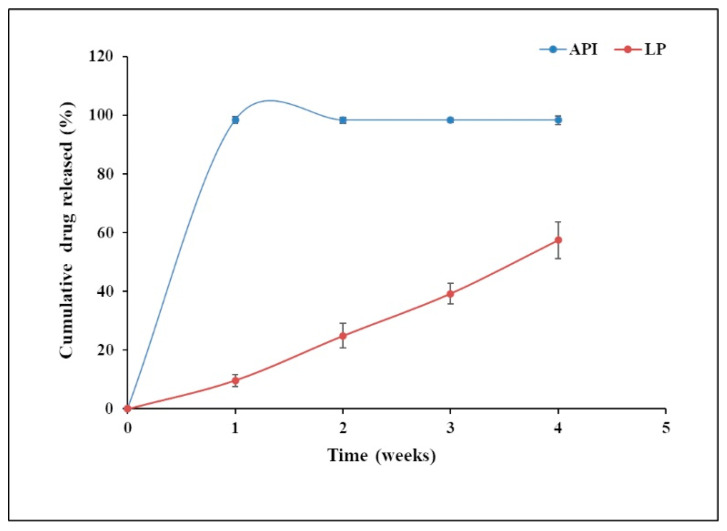
In vitro drug release study of optimized (Batch L6) versus levonorgestrel (API) loaded microneedle.

Figure 4 shows the release profile of liposomes and LNG in the microneedle (LP). In the liposome-loaded microneedle array patch, no initial burst release was observed within 24 h. This may be because of the slow dissolution of LNG, which is sparingly water-soluble, and the release of the LNG from the lipid bilayer. To be released, the LNG has to come out from the lipid bilayer to the surface of the liposomes, and then it will be released. Once the LNG comes into contact with the dissolution medium, the release of the drug takes place. LNG also showed resistance to the highly water-soluble PVA and gelatin matrix. The liposome-loaded microneedle array patch showed 57% drug release in 4 weeks.

The pure LNG (API) drug release profile showed an initial burst release within 24 h. That may be due to the amount of drug that directly underwent dissolution in the dissolution medium, as there was no barrier to the release of the drug. Almost 98% of drug release was observed within 4 weeks, which was similar to results reported earlier in the literature [53].

The patch could be designed for weekly, monthly, quarterly, and annual application based on users’ needs. For this purpose, the dose of the drug, patch size, and the number of microneedles per patch would need to be modified.

### 5.4. In Vivo Study in Rats

**Figure 5:**
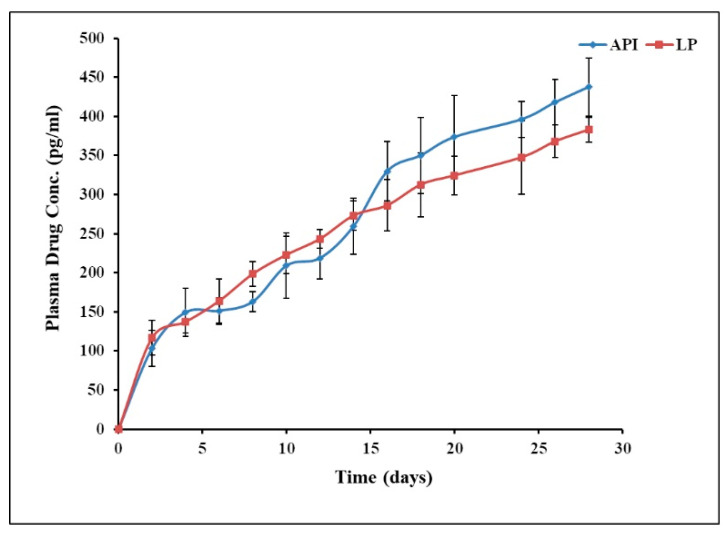
In vivo profile of levonorgestrel liposome-loaded microneedle versus levonorgestrel (API) loaded microneedle.

For further investigation, the behavior of the liposome-loaded microneedle array patch was studied in vivo in rats. After administering the liposome-loaded microneedle and pure drug via a transdermal route, the in vivo behavior was studied in rats (Figure 5).

The liposome-loaded microneedle array patch achieved a concentration above the human therapeutic level (200 pg/mL) in 8 days [54]. It showed an initial burst release within 2 days after application of the patch to the rats and maintained an LNG concentration above the human therapeutic level for more than 1 month. The average amount of drug release was >30 µg/day.

Conversely, pure LNG required 10 days to achieve the human therapeutic level. Similarly to the liposome-loaded microneedle patch, it showed an initial burst release within 2 days. It released more drug and released it faster than the liposome-loaded microneedle.

### 5.5. Mechanical Strength Study

The mechanical strength is an indicator of the skin penetration of the microneedle. Various materials were studied to obtain sufficient mechanical strength. The materials included PEG, PVA, gelatin alone, as well as PVA: PEG (1:1), PEG: gelatin (1:1), and PVA: gelatin (1:1). Finally, PVA: gelatin (1:0.5) resulted in intact needle-shaped microneedles without any bends. It was observed that 1 N strength was sufficient to penetrate through the skin. The graph of mechanical strength is shown in Supplementary Figure S3.

### 5.6. Skin Piercing Study

Piercing microneedles through the skin is essential for the transdermal drug delivery system. To achieve this, the skin-piercing microneedles should have sufficient mechanical strength. The microneedle array patch consisted of an array (18 × 18) consisting of 324 microneedles that were applied onto the rat skin at a speed of 0.1 mm/s. Rat skin is elastic in nature, but as the force increased rapidly, it lost its elasticity. Almost 100% of the microneedles penetrated the skin, as shown in Supplementary Figure S4.

### 5.7. Skin Irritation Study

The microneedle patches were pressed into the dorsal part of the skin for 10 s and then left for 60 s. Then, images of the skin were captured using a camera (Nikon D3500, Japan) using the same conditions (e.g., for light, exposure time, zoom) at 0 and 24 h after application of the patch (Figure S5). The rats tolerated the LNG-loaded microneedle array patch well, with no evidence of redness, inflammation, or swelling to the skin 24 h post patch application. The pain score was calculated using a scale ranging from 0 (no pain) to 10 (hypodermic pain). The pain score was found to be 0, i.e., no pain.

## 6. Conclusions

In the present investigation, liposomes were prepared with a particle size of 157 ± 4.54 nm, polydispersity index of 0.231 ± 0.007, zeta potential of −19 ± 4, and entrapment efficiency of 85%. The cryo HR TEM and cryo FEG SEM analyses showed smooth, spherically shaped particles that correlated with DLS data. The microneedle array patch was developed with a backing membrane, and the microneedles remained implanted under the skin surface after removing the patch. The microneedles were fabricated using a biodegradable polymer that released LNG for >30 days, as shown by in vitro and in vivo study in rats, indicating the promising ability to serve in long-acting contraceptive application. Thus, we concluded that the microneedle array patch would be easy to administer, non-invasive, biodegradable, and suitable for more than 1 month of delivery of the contraceptive agent (LNG).

## Figures and Tables

**Table 1:** Composition of liposome batches.

Ingredients	Batch No					
	L1 (1:5)	L2 (1:7)	L3 (1:10)	L4 (5:5)	L5 (6:4)	L6 (7:3)
Levonorgestrel (mg)	10	10	10	10	10	10
Soya PC (mg)	43.13	60.40	86.26	36.42	40.06	43.12
Oleic acid (mg)	6.87	9.60	13.74	13.58	9.94	6.88
Ethanol (mL)	2.5	2.5	2.5	2.5	2.5	2.5
Phosphate buffer pH 7.4 (mL)	10	10	10	10	10	10

**Table 2:** Characterization of liposome batches.

Batch No.	Particle Size (nm)	Polydispersity Index (PDI)	Zeta Potential (mV)	Entrap. Efficiency (%)
L1	207 ± 4.21	0.216 ± 0.006	−8 ± 2	65.34 ± 5.44
L2	231 ± 6.22	0.249 ± 0.008	−11 ± 3	71.32 ± 3.06
L3	245 ± 7.15	0.338 ± 0.005	−14 ± 3	82.14 ± 4.85
L4	189 ± 5.15	0.315 ± 0.007	−5 ± 1	77.11 ± 6.49
L5	169 ± 6.88	0.305 ± 0.004	−9 ± 2	80.54 ± 3.29
L6	157 ± 4.54	0.231 ± 0.007	−19 ± 4	85.24 ± 6.15

The results are expressed as mean ± SD (n = 3).

## Data Availability

Not applicable.

## Supplementary Materials

The following supporting information can be downloaded at: https://www.mdpi.com/article/10.3390/molecules27072349/s1, Figure S1: Particle size graph of optimized (Batch L6); Figure S2: Microscopy image of microneedle array patch loaded with rhodamine dye; Figure S3: Mechanical behavior of microneedle array under a compression force; Figure S4: Skin penetration study of microneedle array patch after insertion into the rat skin; Figure S5: Skin irritancy study after administration of microneedle.

## Abbreviations

**Term**	**Full Form**
Cryo FEG SEM	Cryo Field Emission Gun Scanning Electron Microscopy
Cryo HR TEM	Cryo High-Resolution Transmission Electron Microscopy
DL	Drug loading
EE	Entrapment Efficiency
HPLC	High-Performance Liquid Chromatography
LNG	Levonorgestrel
LP	Liposomes
MNP	Microneedle Array Patch
PDA	Photodiode array
PS	Particle size
