# Supplementary material for: Levonorgestrel Microneedle Array Patch for Sustained Release Contraception: Formulation, Optimization and In Vivo Characterization
Source: Molecules. 2022 Apr 6;27(7):2349. doi: 10.3390/molecules27072349 (PMC9000369; doi:10.3390/molecules27072349)
Supplement: Supplementary file 1 [file molecules-27-02349-s001.zip › molecules-1580493-supplementary.pdf]

# Supplementary Materials

# Levonorgestrel Microneedle Array Patch for Sustained Release Contraception: Formulation, Optimization and In Vivo Characterization

**Amarjitsing Rajput[a, b*], Riyaz Ali M. Osmani[a, c*], Achyut Khire[a], Sanket Jaiswal[a] and Rinti Banerjee[a]**

1 Nanomedicine Lab, Department of Biosciences and Bioengineering (BSBE), Indian Institute of Technology Bombay (IITB), Mumbai-400076, Maharashtra, India
2 Department of Pharmaceutics, Poona College of Pharmacy, Bharti Vidyapeeth Deemed University, Pune 411038, Maharashtra, India
3 Department of Pharmaceutics, JSS College of Pharmacy (JSSCP), JSS Academy of Higher Education & Research (JSS AHER), Mysuru-570015, Karnataka, India

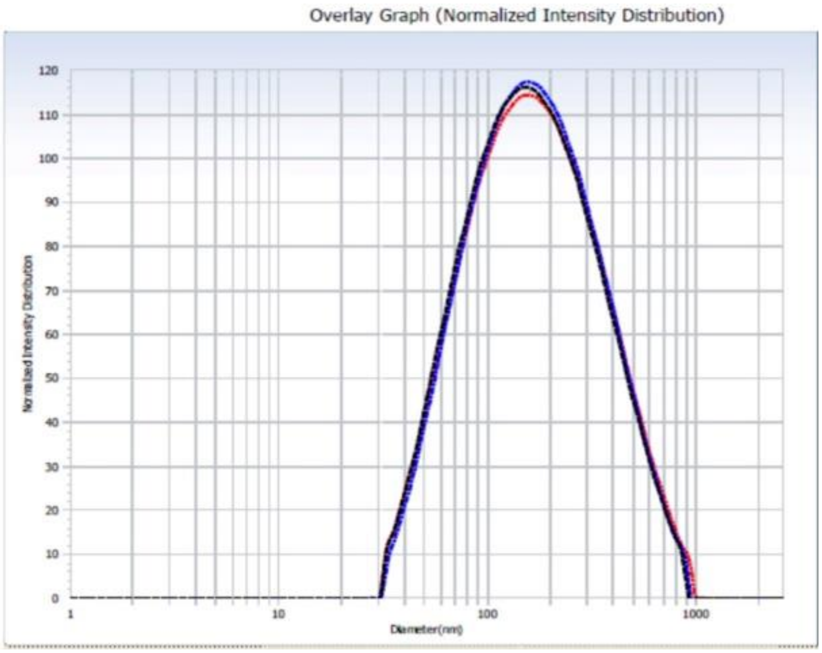


**Figure S1.** Particle size graph of optimized (Batch L6).

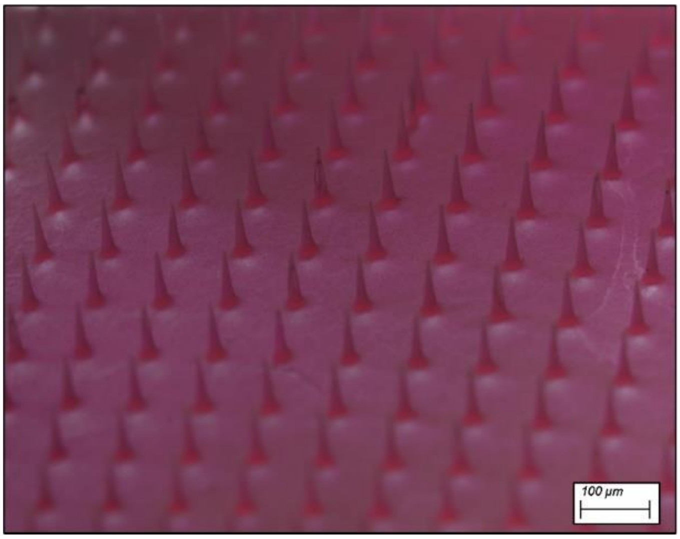


**Figure S2.** Microscopy image of microneedle array patch loaded with rhodamine dye.

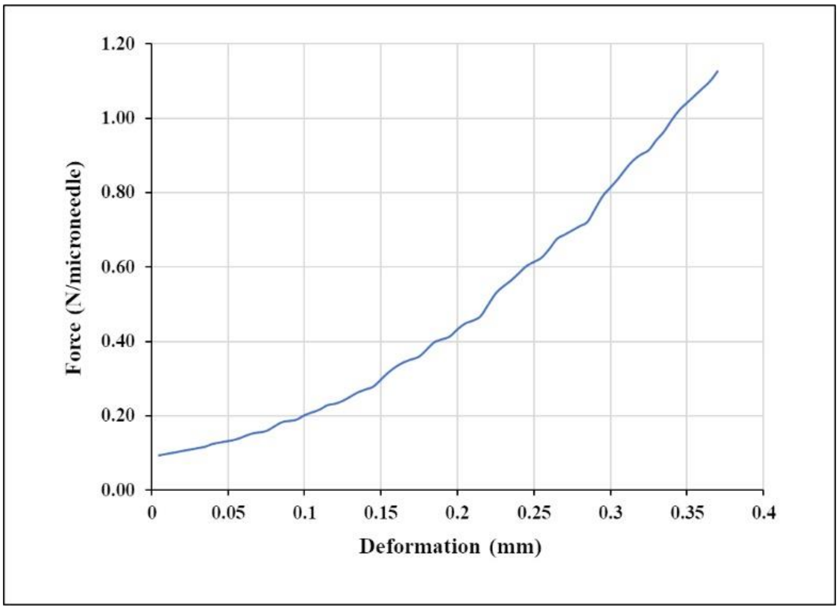


**Figure S3.** Mechanical behavior of microneedle array under a compression force.

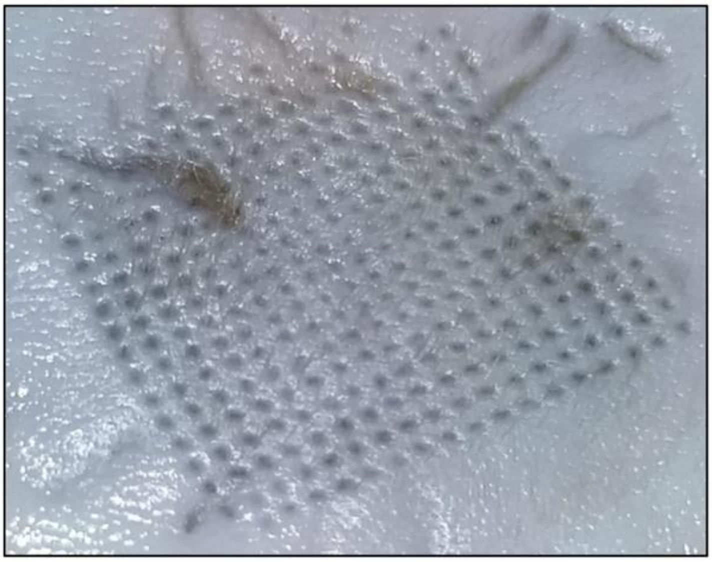

**Figure S4.** Skin penetration study of microneedle array patch after insertion into the rat skin.

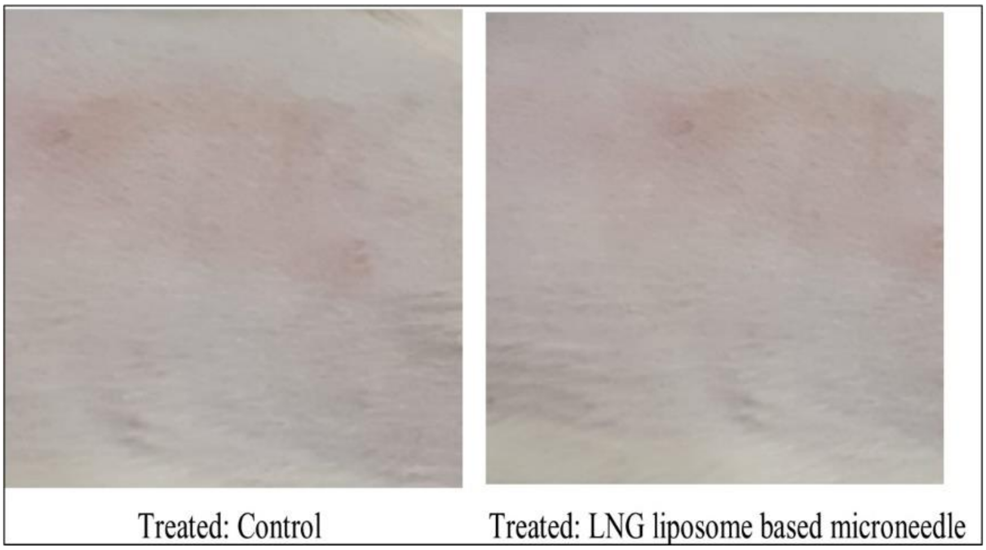


**Figure S5.** Skin irritancy study after administration of microneedle.
